# Development of Nanocoated Filaments for 3D Fused Deposition Modeling of Antibacterial and Antioxidant Materials

**DOI:** 10.3390/polym14132645

**Published:** 2022-06-29

**Authors:** Turdimuhammad Abdullah, Rayyan O. Qurban, Mohamed Sh. Abdel-Wahab, Numan A. Salah, Ammar AbdulGhani Melaibari, Mazin A. Zamzami, Adnan Memić

**Affiliations:** 1 Center of Nanotechnology, King Abdulaziz University, Jeddah 21589, Saudi Arabia; 1202908@gmail.com (T.A.); mshabaan90@yahoo.com (M.S.A.-W.); nsalah@kau.edu.sa (N.A.S.); aamelaibari@kau.edu.sa (A.A.M.); 2 Department of Biochemistry, Faculty of Science, King Abdulaziz University, Jeddah 21589, Saudi Arabia; rayyanqurban@outlook.com (R.O.Q.); mzamzami@kau.edu.sa (M.A.Z.); 3 Department of Mechanical Engineering, Faculty of Engineering, King Abdulaziz University, Jeddah 21589, Saudi Arabia

**Keywords:** FDM, PBS, lignin, Ag/ZnO, antioxidant, antimicrobial

## Abstract

Three-dimensional (3D) printing is one of the most futuristic manufacturing technologies, allowing on-demand manufacturing of products with highly complex geometries and tunable material properties. Among the different 3D-printing technologies, fused deposition modeling (FDM) is the most popular one due to its affordability, adaptability, and pertinency in many areas, including the biomedical field. Yet, only limited amounts of materials are commercially available for FDM, which hampers their application potential. Polybutylene succinate (PBS) is one of the biocompatible and biodegradable thermoplastics that could be subjected to FDM printing for healthcare applications. However, microbial contamination and the formation of biofilms is a critical issue during direct usage of thermoplastics, including PBS. Herein, we developed a composite filament containing polybutylene succinate (PBS) and lignin for FDM printing. Compared to pure PBS, the PBS/lignin composite with 2.5~3.5% lignin showed better printability and antioxidant and antimicrobial properties. We further coated silver/zinc oxide on the printed graft to enhance their antimicrobial performance and obtain the strain-specific antimicrobial activity. We expect that the developed approach can be used in biomedical applications such as patient-specific orthoses.

## 1. Introduction

Over the past decade, digital fabrication technologies developed using three-dimensional (3D) printing have experienced exponential growth [1,2]. Driven by the possibility to generate highly customizable products, 3D printing has found numerous applications ranging from healthcare to automotive and even agriculture industries [3,4,5]. The possibility to fabricate such customized products using 3D printing (also known as additive manufacturing) is based on the process of sequential addition of materials [1,6,7]. Each material layer is derived from a geometrical representation of the desired object that is often rendered using computer-aided design (CAD) drawings and software [8]. Such approaches provide several advantages but most notably they support rapid prototyping and on-demand manufacturing of products [9]. These quick design-product cycles allow for fast optimization of product properties [9,10]. Similarly, multi-material objects can be produced, yielding the opportunity for even better tuning of product properties, including various physical, chemical, and biological parameters as required by specific applications [9,11,12].

There have been several 3D-printing methods developed for various applications, some with distinct properties and advantages [13]. However, one of the first 3D-printing approaches developed in the 1990s was based on fused deposition modeling (FDM), also known as fused filament fabrication (FFF) [14]. Generally, in FDM, a thermoplastic polymeric material (i.e., a filament) is heated above its melting temperature to be extruded through a nozzle that can be deposited in a layer-by-layer process to fabricate a designed object [13,15]. Although other approaches could offer some advantages in terms of speed or print resolution, currently, FDM has primarily been applied in several biomedical-type settings, including 3D printing of medical devices and instruments [16,17,18]. For example, FDM prints derived from patient computed tomography (CT) or magnetic resonance imaging (MRI) scans could be used for improved preoperative planning or printing patient-specific orthoses and bracing [16,19]. However, not all thermoplastic polymers demonstrate proper processability and 3D printability [6,20]. Therefore, to propel further biomedical applications, there is a pressing need to develop low-cost and advanced printable filaments [20].

One thermoplastic polymer that has recently gained a lot of attention is polybutylene succinate (PBS) [21,22]. PBS is an aliphatic polyester that exhibits similar properties to polypropylene (PP) that is amenable to being processed by extrusion, injection molding, and 3D printing [22,23]. PBS also has several advantages and could be used to replace PP, especially in 3D printing of patient-specific orthoses [22]. For example, PBS exhibits a high degree of crystallinity, cell-friendly surface characteristics, and biodegradability [22]. Other advantages of PBS include exceptional plasticizing effects for mechanically stiff but brittle polymers [21,24]. Nevertheless, PBS lacks antibacterial effects; therefore, it is best to combine it with other natural polymers that could improve its bulk properties [1,25]. One low-cost polymer, lignin, contains both methoxyl and phenolic hydroxyl groups that make it a potent antioxidant and antimicrobial agent [26]. Furthermore, it is plentiful, renewable, and significantly underutilized [27]. In addition, lignin exhibits several other key properties, including biocompatibility, 3D-printing processability, and printability. Adding lignin into a host polymer could therefore improve the bulk antimicrobial properties of 3D-printed structures while not affecting the biological properties of the composite [15].

Another way to further improve the antimicrobial properties of 3D-printed objects is by depositing an antimicrobial layer onto their outer surface [28]. Several surface-coating methods have been developed, including wet chemical (i.e., dip-coating) and vacuum deposition methods (i.e., various sputtering approaches). Both metal (i.e., silver) and metal oxide (i.e., zinc oxide, copper oxide, etc.) nanocoatings have shown much promise in preventing pathogen growth [1,29]. Such bulk objects could be used for 3D printing of protective equipment, hospital tables, implants, and patient-specific orthoses [30]. In addition to the choice of the nanocoating itself, another important consideration is the change in surface topography and roughness as a result of the coating [28]. The choice of nanocoating and its resulting properties (i.e., hydrophobicity, steric hindrance, and noncovalent interactions) have been shown to affect the attachment and formation of biofilms. Therefore, by combining various polymer blends, it might be possible to template and control the properties and effectiveness of individual or a combination of nanocoatings. These optimized and controlled nanocoatings might provide improved or even strain-specific antibacterial activity [31,32]. Such scaffolds could propel utility torward several biomedical applications.

In our current work, we fabricated multifunctional filaments for biomedical applications, including the usage as patient-specific orthoses. First, we coherently mixed the filament precursors (i.e., lignin with PBS polymer) by solvent casting. Next, we optimized the extrusion parameters such as extrusion speed and temperature to produce filaments with uniform and consistent diameters. We then examined the biophysical characteristics of lignin–PBS filaments, such as their surface morphology, rheological properties, print fidelity as well as antioxidant properties. Next, we nanocoated zinc, silver, or their combination onto filament prints using RF sputtering. Finally, we evaluated the antimicrobial properties of these nanocoated filaments against several pathogens that would be relevant to patients wearing orthotic braces for prolonged periods.

## 2. Materials and Methods

### 2.1. Materials

Polybutylene succinate (PBS, MW = 97,600) was acquired from Showa Denko, Tokyo, Japan. Lignin (Biolignin™, CIMV, Labège, France) was provided by Nanoscience Centre (MAVI, Aprilia, Italy). Chloroform was purchased from Sigma-Aldrich (St. Louis, MO, USA). Silver (Ag, 99.999% purity), and zinc oxide (ZnO, 99.999% purity) were obtained from Lesker (Dresden, Germany). The suppliers and specifications of other chemicals are mentioned elsewhere.

### 2.2. Preparation of Filament and 3D Printing

The solvent-casting method was applied to homogeneously mix PBS with lignin prior to extrusion. The different mass fractions of lignin dispersed in chloroform (100 mL for 30 g of composite) by sonication for 30 min, then PBS pellets were added gradually into the dispersion under magnetic stirring. Afterward, the container was covered and kept at a stirrer at 90 °C until PBS was completely dissolved. Finally, the solution was cast on a large steel pan by evaporating the solvent at 120 °C. The filament extrusion was conducted in a homemade desktop filament extruder with 1.75 mm of the nozzle. The solvent-casted composites were pelletized before feeding into the extrusion. The effect of the extrudate temperature and the motor speed was studied to optimize the thickness and uniformity of the filament. A water bath is placed next to the extrudate to cool down and collect the filament. The produced filament was printed in a Robo E3 FDM printer (Robo3D, San Diego, CA, USA). The predesigned Standard Tessellation Language (STL) file was sliced by Robo 4.3.2v software ((Robo3D, San Diego, CA, USA). The printing temperature was fixed at 110 °C, the printing speed was 60 mm/s, and the migration speed was 80 mm/s. The layer height was set at 0.12, bottom/top, and the shell thickness was set at 1.2 mm. Square/line patterns were used for infilling, and the filling ratio was determined according to the desired application of the printed item. For example, the filling ratio of the sample designed for the rheology test was 100%.

### 2.3. Sputter Coating

Coating of Ag (ZnO, and their combination on the 3D-printed composites was performed in a radio frequency (RF) magnetron sputtering system (Syskey Technologies, Hsinchu, Taiwan). The generation of the plasma was achieved by introducing 20 standard cubic centimeters per minute (SCCM) of argon gas with a flow rate at 200 watts of RF power. The base pressure was fixed at 9 × 10^−6^ Torr, while 5 × 10^−3^ Torr of operating pressure was used. 15 rpm of substrate rotation speed, 14 cm of target–substrate distance, and 600 s of deposition time were applied to create a nanocrystalline 100 nm of thin layer on the printed composite.

### 2.4. Characterization

The melting and crystallization temperature of the PBS/lignin composites were determined according to differential scanning calorimetry (DSC) measurement using DSC-60 (Shimadzu Corporation, Kyoto, Japan). In brief, 10 mg of printed composites loaded on an aluminum pan were heated up to 150 °C at 5 °C/min and were then cooled down to 30 °C at 5 °C/min under a nitrogen atmosphere. The surface micrographs of the printed composites were observed by JSM 7600F scanning electron microscopy (FESEM, JEOL, Tokyo, Japan). The images were recorded at an acceleration voltage of 15 kV through a lower secondary electron detector (LEI). The working distance was different for each sample in the process of surface focusing and image optimization.

The temperature-dependant rheological behavior of the printed composites was determined by an oscillatory temperature-sweep test using a Discovery Hybrid Rheometer (DHR-3, TA instrument, New Castle, DE, USA). The composites were printed in a cylindrical shape with 1000 µm of thickness and 40 mm of diameter and inserted between a parallel plate. Then a temperature in the range of 100~150 °C is applied to the sample while fixing the oscillation frequency at 10 rad·s^−1^ and oscillatory strain at 2%. Next, an oscillatory amplitude-sweep test in the range of 0.002~20% of oscillatory strain was conducted for the printed composites at 110 °C and 10 rad·s^−1^.

### 2.5. Antibacterial and Antioxidant Performance

The agar diffusion method was applied to assess the antimicrobial performance of the samples. Five different colonies of ATCC bacteria and fungi were collected from King Abdulaziz Hospital. The needle-drop size of colonies was immersed in Lysogeny broth (LB) media containing 100 μg/mL of ampicillin and cultured at 37 °C overnight. Then 200 μL of cultured strains were evenly diffused on the plate surface of ampicillin-contained LB-agar (100 μg/mL) to obtain a mat of bacteria. Afterward, the samples in the shape of a flat circle (5 mm diameter, 1 mm thick) in triplicates were added, distributed regularly, and numbered according to the concentration of lignin and nanoparticles added to them. Finally, the microbial strain was incubated overnight at 37 °C.

The total antioxidant capacity (TAOC) of the composites was determined by the Ferric Reducing Ability of Plasma (FRAP) assay. The assay kit was purchased from Beijing Solarbio Science & Technology Co., Ltd. (Beijing, China), and measurements were conducted according to the manufacturer’s instructions. The TAOC data for the composites were further expressed as Vitamin C Equivalent Antioxidant Capacity (VCEAC). Other details regarding the measurement can be found in our previous work [26].

## 3. Results and Discussion

**Figure 1:**
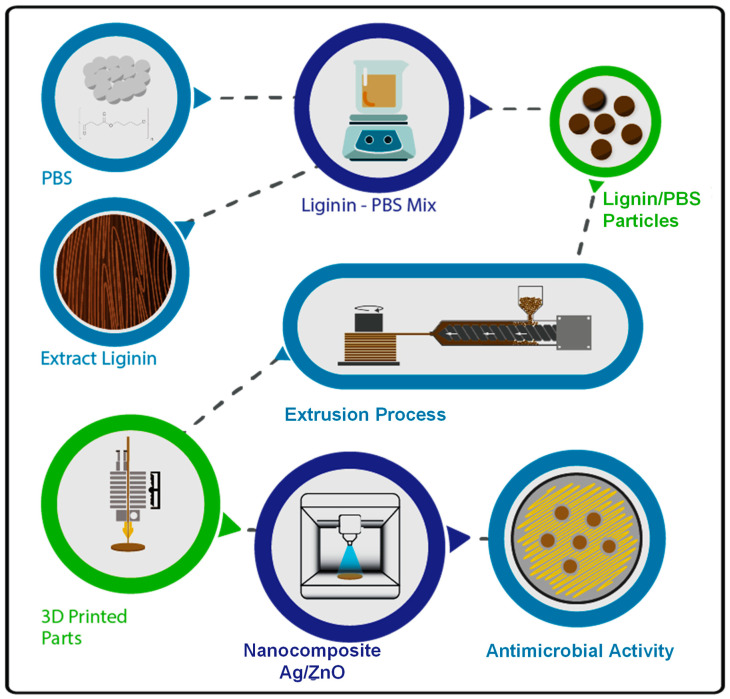
The schematic presentation of the creation of nanocoated PBS/lignin composite grafts by FDM printing and RF sputtering.

In this study, we successfully printed PBS/Lignin composites with different mass ratios using FDM, which were further coated with Ag/ZnO by an RF sputtering (Figure 1). We applied the solvent-casting method to uniformly disperse submicron lignin particles within PBS, and then the PBS/lignin mixture was extruded using a custom-made single-screw extrusion system in the form of filaments. Considering that most of the currently available desktop FDM printers support the 1.75 mm filament diameter with uniform thickness, we used the nozzle with the same diameter and immediately cooled down the filament with cold water to avoid stretching and thinning of the molten composite [33]. However, controlling the feed rate and the extrusion temperature has been suggested to be critical in maintaining the desired thickness of the filaments [34]. Therefore, we first studied the effects of these two parameters on the thickness and uniformity of PBS filament. As shown in Figure S1, the thickness of the PBS filament is higher than 2 mm when the nozzle is heated up just above the melting point of PBS. The filament became gradually thinner with increased extrusion temperature and the feed rate, and optimum thickness was obtained at 110 °C and 4 kg/h of feed rate. Accordingly, we applied this condition to fabricate the composite filament with 0.5, 1.5, 2.5, and 3.5% of lignin submicron particles.

**Figure 2:**
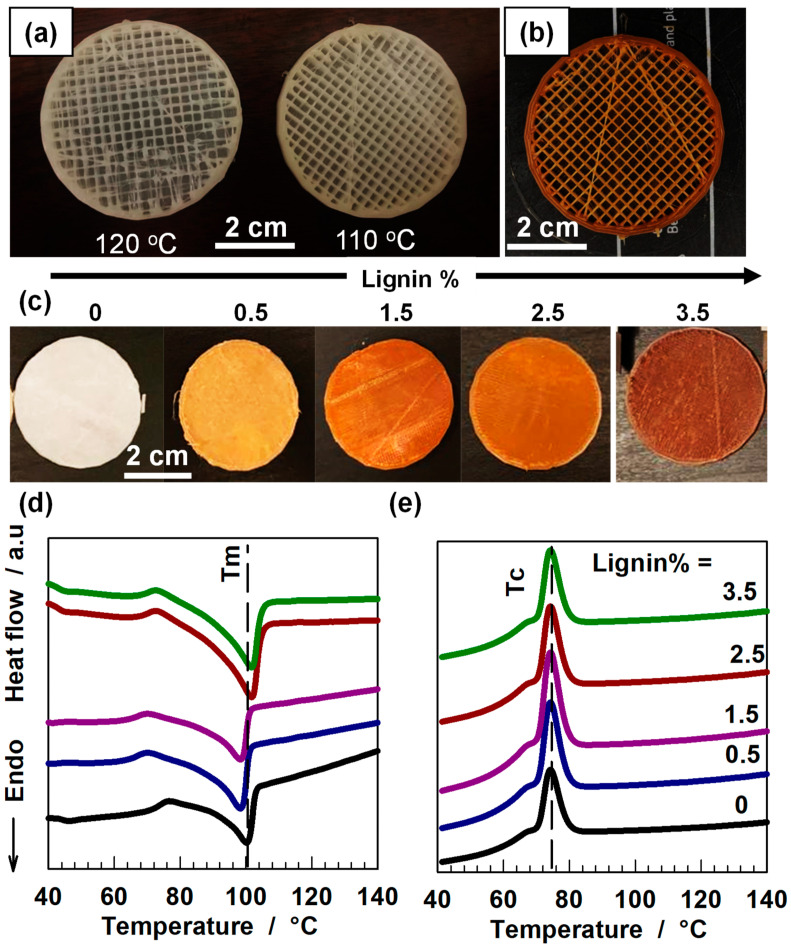
(**a**) Printing quality of PBS at different extrusion temperatures. (**b**) the printing quality of the composite with 2.5% lignin at 110 °C. The original CAD was designed as a circular shape within square networks. (**c**) The appearance of printed composites with different mass fractions of lignin. (**d**,**e**) DSC traces of the PBS/lignin composites during heating (**d**) and cooling (**e**).

Afterward, we assessed the printability of the generated filaments and noticed that the extrusion temperature was critical to obtaining high-quality printing. We designed a circular network by computer-aided design (CAD) connected by square shapes and printed them using PBS filament at different temperatures. When the extrusion temperature was at 120 °C, many errors were observed in the structure of the printed item (Figure 2a). For instance, many square shapes were not clearly present, and numerous undesirable beads can be seen in the figure. These errors can be greatly minimized by decreasing the extrusion temperature to 110 °C. The incorporation of lignin further improved the printing quality of PBS, particularly the PBS/lignin composite with 2.5% of lignin showed the highest printing quality among the studied samples (Figure 2b). Thereupon, we found that the addition of lignin within PBS results in uniform color changes in the printed sample. The composite with 0.5% of lignin exhibited a light brown color, and the increased mass fraction of lignin darkened the color of the printed composite (Figure 2c). The temperature-dependent phase-transition behavior of the printed composite was studied by DSC. DSC is an analytical tool to examine the fusion and crystallization behavior of thermoplastic-based composite materials. It is critically important to select proper extrusion and deposition conditions during FDM printing. All the printed composites showed an endothermic and an exothermic peak at 100 and 77 °C, respectively (Figure 2d,e). These peaks correspond to the melting and crystallization temperature of PBS.

**Figure 3:**
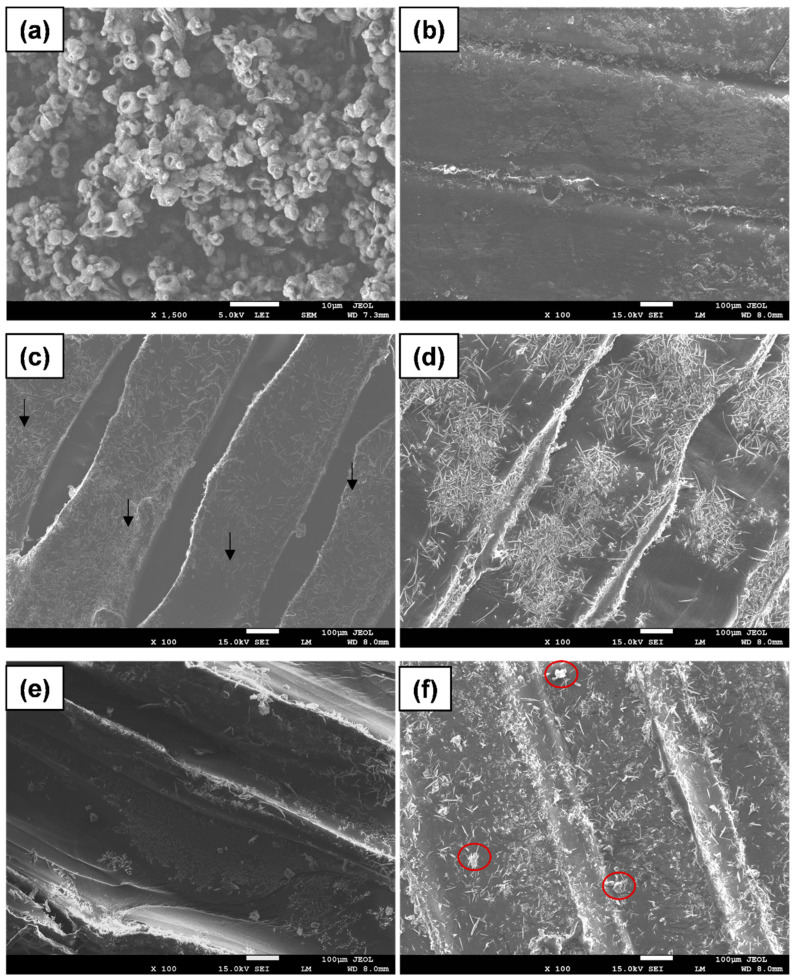
SEM micrograph of lignin (**a**), PBS (**b**) and PBS/lignin composites with 0.5 (**c**), 1.5 (**d**), 2.5 (**e**), and 3.5 (**f**) % of lignin. The arrows show well-dispersed lignin particles, while the red circles show agglomerated lignin particles.

The presence of lignin in the printed composite was confirmed via SEM micrography (Figure 3). Lignin submicron particles could be identified from the SEM image, even though the mass fraction of lignin is as low as 0.5%. The density of lignin particles increases with increased lignin content. However, agglomeration of the particles started to occur when the mass fraction of lignin was above 2.5%. A similar phenomenon was observed in our previous work that the dispersion of lignin within the polymeric network is more dispersed when the amount of lignin is lower [26].

**Figure 4:**
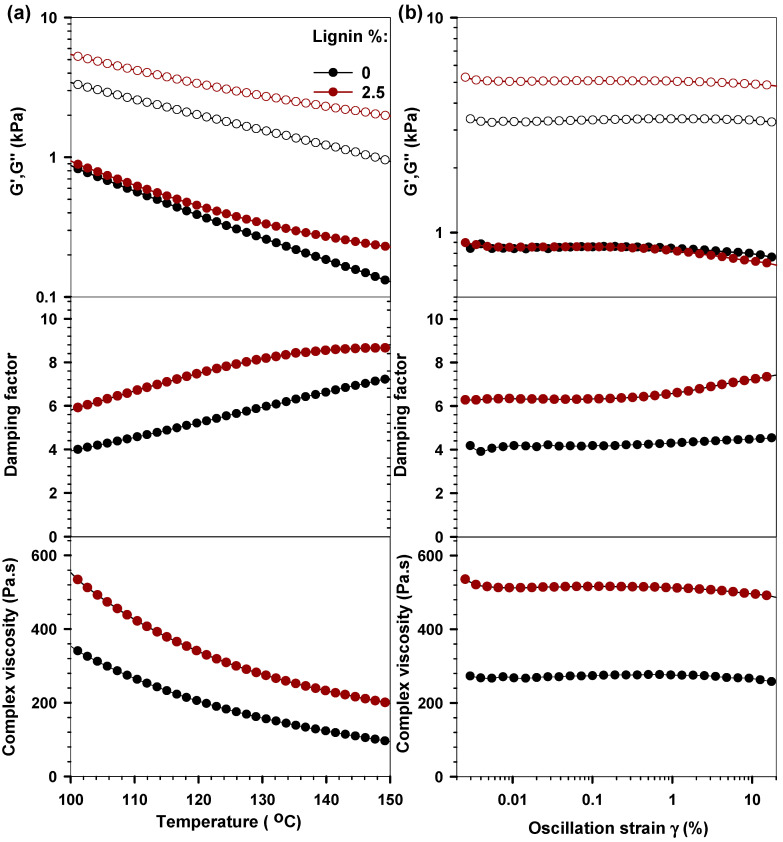
(**a**) The temperature-dependent variation of storage modulus, loss modulus, damping factor, and complex viscosity of pure PBS and composite with 2.5% lignin. (**b**) The oscillatory strain-dependent variation of storage modulus, loss modulus, damping factor, and complex viscosity of pure PBS and composite with 2.5% lignin at 110 °C.

Next, we studied the rheological behavior of the PBS/lignin composite by an oscillatory temperature-sweep test above its melting temperature. Generally, the viscous modulus of both composite and pure PBS was 4~8-times higher than their storage modulus at the studied temperature range, which reveals their clear liquid-like characteristics above their melting point (Figure 4a). With the incorporation of lignin-enhanced dynamic moduli of PBS, we noticed a significant increase in the viscous modulus. For instance, the viscous modulus of the composite (5.42 kPa) is 1.6-times higher than that of pure PBS (3.4 kPa) at 100 °C. As a result, the damping ratio and the complex viscosity of the composite were found to be higher than that of pure PBS. We also noticed that the damping ratio increases, and complex viscosity decrease within the increased temperature. Furthermore, the viscosity of the composite is proportional to the mass fraction of lignin, which could be due to the nucleating effects of lignin (Figure S2) [35].

We further evaluated the viscoelastic characteristics of the composite at 110 °C via an amplitude-sweep oscillatory test (Figure 4b). Both PBS and PBS/lignin composite generally showed typical linear viscoelastic characteristics in the studied oscillatory strain range (0.002~20%). This result indicates that a large range of amplitude can be applied to the PBS/lignin composite without destroying its structure. Previous studies suggest that the material that can exhibit a longer elastic response (up to 10% oscillatory strain or more) could be printed by FDM with high quality [15]. Additionally, the composite with 2.5% lignin showed viscosity (approximately 550 Pas) at 110 °C in the studied amplitude strain range. This value is comparable to the complex viscosity of Acrylonitrile Butadiene Styrene (ABS) at its most suitable printing temperature (i.e., at 250 °C) [36]. Overall, the incorporation of lignin improved the viscoelastic properties of PBS, which further enhanced its printing quality. The hydrodynamic interaction formed between PBS and lignin submicron particles could be the main reason for this enhancement.

**Figure 5:**
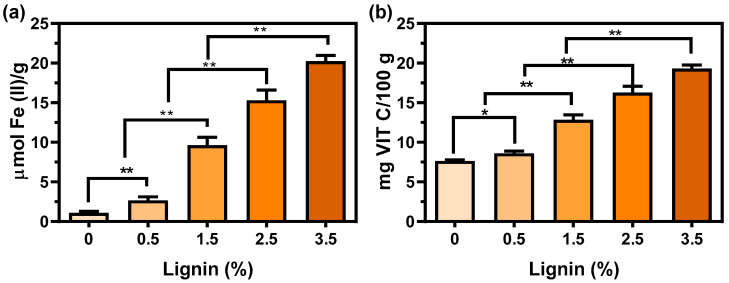
(**a**) The ferric reducing ability of plasma (FRAP) value for the printed composites, and (**b**) The vitamin C equivalent antioxidant activity of the composites. (*n* = 3).

Next, we evaluated the effect of lignin addition on the antioxidant properties of PBS. A ferric-reducing ability of plasma (FRAP) method was utilized to measure the antioxidant capacity of the printed lignin/PBS composite and the obtained value was compared with the vitamin C equivalent antioxidant capacity (VCEAC). The correlation equation between FRAP and VCEAC can be found elsewhere [26]. Vitamin C is a commonly suggested antioxidant compound in biomedical applications [37,38], and this protocol has been found to be highly efficient for measuring the antioxidant capacity of plant-derived phenolic chemicals [38]. As shown in Figure 5, the antioxidant capacity of the printed samples increased with the mass fraction of lignin. For instance, 19.3 mg/100 VCEAC was obtained for PBS/lignin with 3.5% of lignin, which is approximately 2.5 times higher than pure PBS. The increased antioxidant capacity is mainly due to the presence of hydroxyl and methoxyl functional groups in lignin which could donate hydrogen to stabilize the free radicals [39]. Moreover, the literature also suggested that lignin has the ability to constrain the related enzymes from encouraging the production of the free radical [40].

Finally, we assessed the inhibition effects of the printed composite against five different microorganisms. Among them, *Escherichia Coli (E.coli*, ATCC 25922) and *Pseudomonas Aeruginosa* (*P. aeruginosa,* ATCC 27853), and *Klebsiella Aerogenes* (*K. aerogenes,* ATCC 13048) are Gram-negative bacteria. *Staphylococcus Aureus* (*S. aureus*, ATCC 29213) is a Gram-positive bacterium. These are some of the most common multidrug-resistant bacteria and are responsible for many nosocomial infections [41,42]. We also examined the antifungal activity of the printed samples using *Candida Albicans* (*C. Albicans*, ATCC 14053) as a representative [43]. We found that PBS/lignin composite alone is not enough to exhibit significant antimicrobial activity against these pathogens (Figure S3). Therefore, we coated Ag, ZnO, and their mixture on the printed composite to improve their antimicrobial performance.

**Figure 6:**
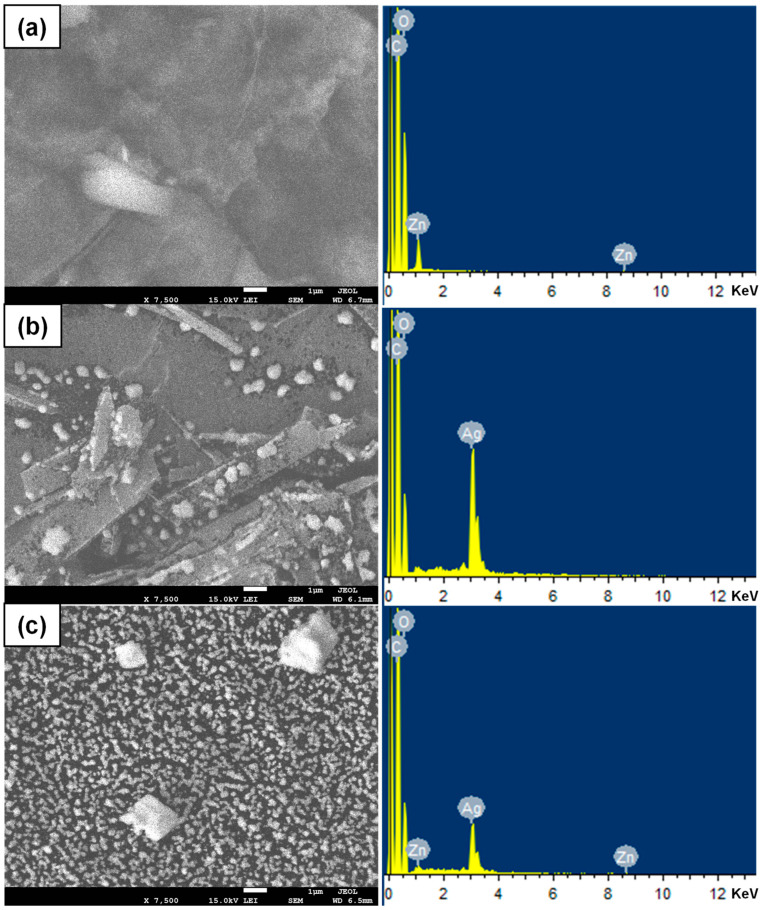
The SEM micrograph and the corresponded EDX spectra of the composite after being coated by ZnO (**a**), Ag (**b**), and their mixture (**c**).

The surface morphology and the corresponding EDX spectra of the composite after being coated by Ag, ZnO, and their mixture are shown in Figure 6. Compared to ZnO and Ag, dispersion of Ag/ZnO onto the composite is more uniform, where the size of the nanostructure is much smaller than that of ZnO and Ag. This could be because the co-sputter coating of two different materials simultaneously could prevent the agglomeration of the dispersed nanoparticles [44]. The EDX spectra of the sputter-coated composites confirm the presence of zinc, silver, oxygen, and carbon. The corresponding peaks appeared at 1 and 8.54 KeV for zinc, 3.5 KeV for silver, 0.5 KeV for oxygen, and 0.27 KeV for carbon, respectively.

**Figure 7:**
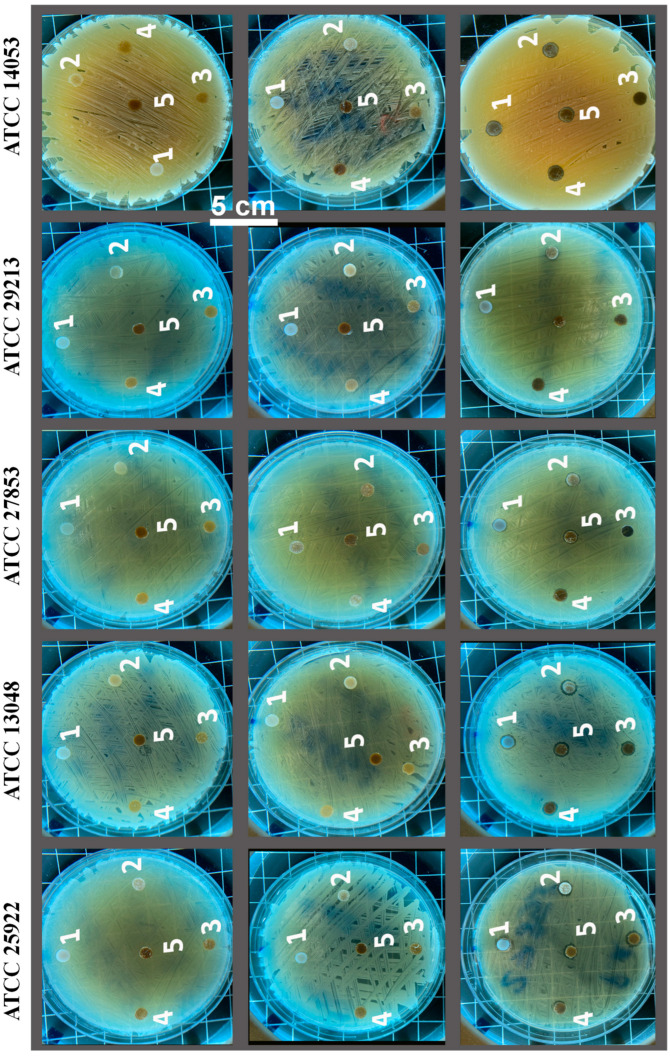
Representative inhibition zone of the composites coated by ZnO (**left**), Ag (**middle**), and Ag/ZnO (**right**) against *Escherichia Coli* (ATCC 25922), *Klebsiella Aerogenes* (ATCC 13048), *Pseudomonas Aeruginosa* (ATCC 27853), *Staphylococcus Aureus* (ATCC 29213), and *Candida Albicans* (ATCC 14053). The coded number 1–5 in the disk represents 0% (1), 0.5% (2), 1.5% (3), 2.5% (4), and 3.5% (5) of lignin in the composite.

The antimicrobial performances of the PBS/lignin composites after being coated by Ag, ZnO, and Ag/ZnO are presented in Figure 7. Interestingly, the coating by Ag and ZnO alone did not notably enhance the antimicrobial properties of the printed composites, whereas the printed samples coated by Ag/ZnO showed different levels of inhibition zone against the studied pathogens depending on the lignin content. In general, a higher mass fraction of lignin in the composite provided a larger inhibition zone compared to the lower-mass-fraction one (Figure S4). Particularly, the 3D-printed composite grafts within 3.5 wt.% of lignin coated by Ag/ZnO showed a clear inhibition zone against all the studied microorganisms. This result suggests that it is possible to create the synergetic effect between multiple antimicrobial agents, so the fabricated materials can be resistant to invasions of a broad variety of microbes [45,46]. We hypothesize that the PBS/lignin blend (i.e., increasing lignin content) plays a role in templating the deposition of Ag/ZnO-nanocoated layer in such a way that it gives rise to its synergistic antibacterial effects. Particularly, sparse dispersion of Ag/ZnO (i.e., as nanoparticles without agglomeration), as shown in Figure 6, could facilitate the improved release of metal ions and enhance the overall antimicrobial activity of the composites [1]. Furthermore, we are not the first to report on the synergism between Ag and ZnO antimicrobial activity; several groups have reported similar findings [47,48,49]. For example, Bednář et al., showed a positive synergistic antimicrobial effect between Ag and ZnO. They attributed the synergistic effect to the Ag/ZnO nanohybrid-enhanced surface area and improved adsorption capacity to the surface of different microorganisms [47]. On the other hand, Ghosh et al. attributed this synergism to an improved electrostatic interaction between the Ag/ZnO nanohybrids and the microbial cell wall and their cell permeability [48]. We hypothesize that it could be a combination of both effects; however, these studies are beyond the current scope and are planned for future research.

## 4. Conclusions

We successfully printed a series of PBS/lignin composites via the FDM approach and evaluated their antioxidant and antimicrobial performance. The extrusion temperature is found to be critical to determining the printability of the composites, and 110 °C extrusion temperature showed the best printing quality. The incorporation of lignin improved the printing quality, dynamic moduli, and antioxidant performance of PBS. The antimicrobial activity of the 3D-printed composite grafts can be enhanced by nanocoating with Ag/ZnO, and it is proportional to the lignin content. Overall, scaffolds with the highest amount of lignin coated by both Ag and ZnO showed antimicrobial activity against five different microorganisms. Overall, these nanocoated, 3D-printed PBS/lignin composites could be potentially used as patient-specific orthoses.

## Data Availability

The data presented in this study are available on request from the corresponding author.

## Supplementary Materials

The following are available online at https://www.mdpi.com/article/10.3390/polym14132645/s1. Figure S1. Fabrication of the 3D printing filament at different extrusion temperatures and 5 kg/h (a), 4 kg/h (b), and 3 kg/h (c) of feed rate. Figure S2. The temperature-dependent variation of complex viscosity of composite with different lignin mass fraction. Figure S3. Representative inhibition zone of the non-coated composites against 5 different microorganisms. Figure S4. Inhibition zone of the composites with different mass fractions of lignin coated by Ag/ZnO against *Escherichia coli*.
