# Supplementary material for: Development of Nanocoated Filaments for 3D Fused Deposition Modeling of Antibacterial and Antioxidant Materials
Source: Polymers (Basel). 2022 Jun 29;14(13):2645. doi: 10.3390/polym14132645 (PMC9269528; doi:10.3390/polym14132645)
Supplement: Supplementary file 1 [file polymers-14-02645-s001.zip › polymers-1757841-supplementary.pdf]

polymers MDPI

Supplementary materials

Development of Nanocoated Filaments for 3D Fused Deposition Modeling of Antibacterial and Antioxidant Materials

Turdimuhammad Abdullah[1], Rayyan O. Qurban[2], Mohamed Sh. Abdel-Wahab[1], Numan A. Salah[1], Ammar AbdulGhani Melaibari[1], Mazin A. Zamzami[2], and Adnan Memić [1,*]

1 Center of Nanotechnology, King Abdul Aziz University, Jeddah 21589, Saudi Arabia; 1202908@gmail.com (T.A.); rayyanqurban@outlook.com (R.O.Q.); mshabaan90@yahoo.com (M.S.A.); nsalah@kau.edu.sa (N.A.S.); aamelaibari@kau.edu.sa (A.A.M.); amemic@kau.edu.sa (A.M);
2 Department of Biochemistry, Faculty of Science, King Abdulaziz University, Jeddah, Saudi Arabia; mzamzami@kau.edu.sa (M.Z.)
3 Department of Physics, Faculty of Science, King Abdulaziz University, Jeddah, Saudi Arabia
4 Department of Medical Laboratory Technology, Faculty of Applied Medical Sciences, King Abdulaziz University, Jeddah, Saudi Arabia
5 Department of Electrical and Computer Engineering, Faculty of Engineering, King Abdulaziz University, Jeddah, Saudi Arabia
* Correspondence: amemic@kau.edu.sa

Received: date; Accepted: date; Published: date

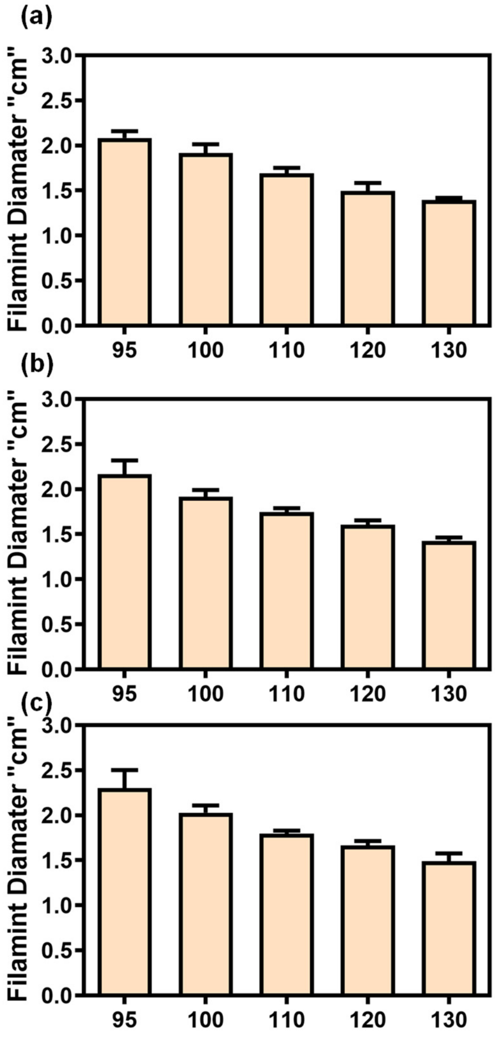

Figure S1. Fabrication of the 3D printing filament at different extrusion temperature and 5 kg/h (a) , 4 kg/h (b), and 3 kg/h (c) of feed rate.

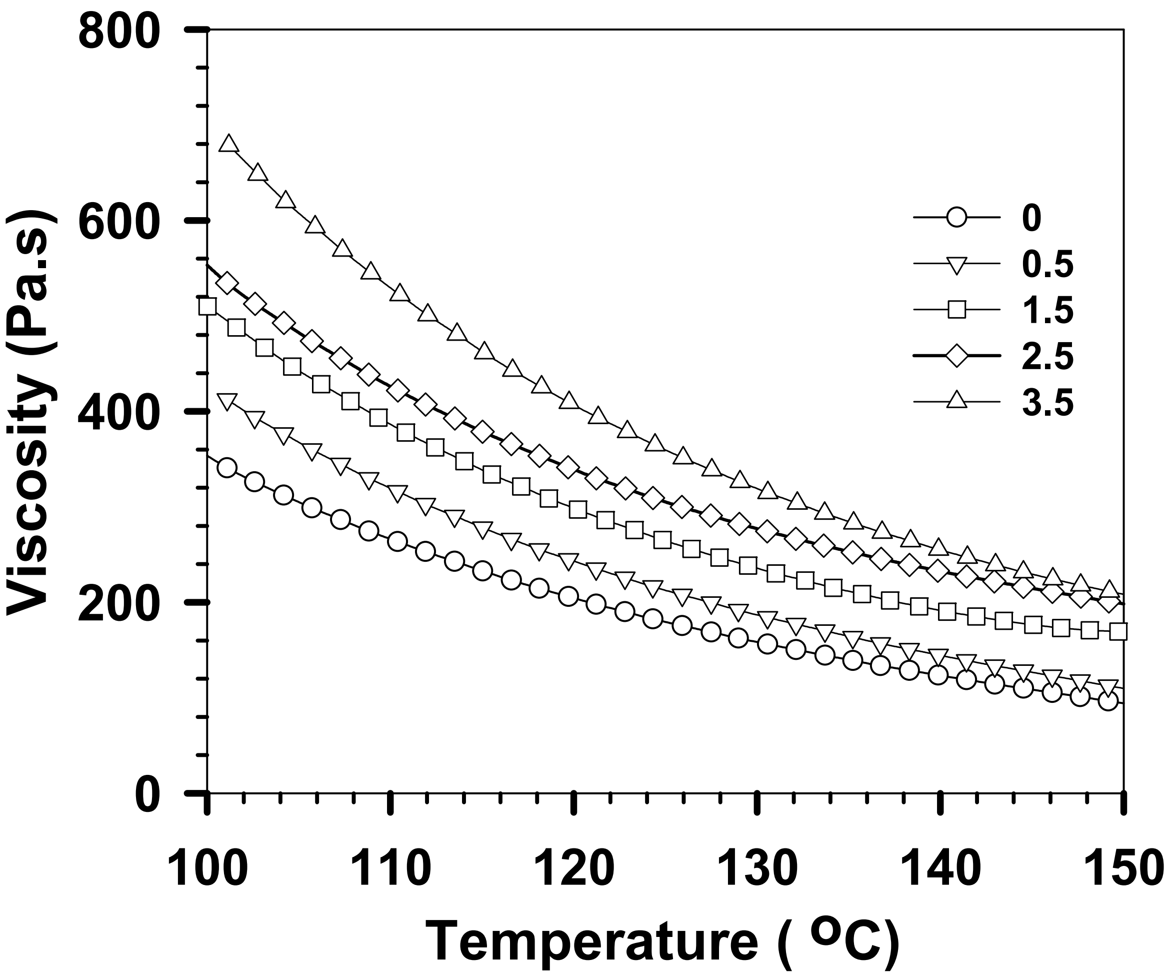

Figure S2. The temperature-dependent variation of complex viscosity of composite with different lignin mass fraction.

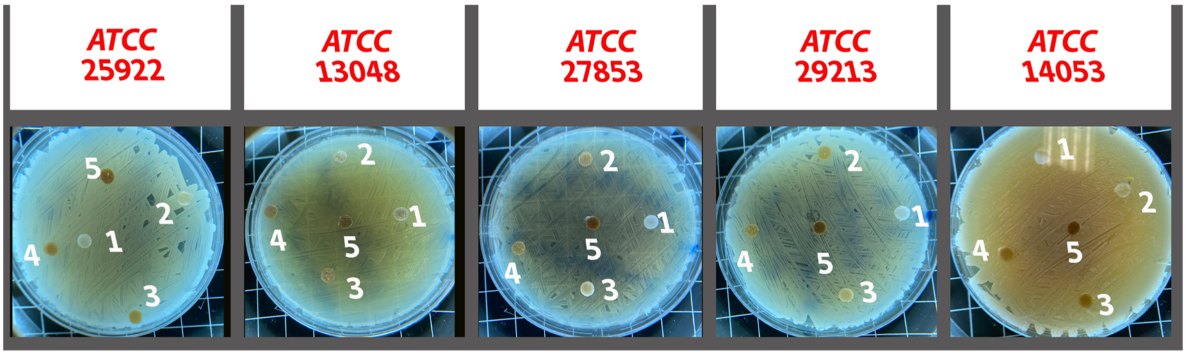

Figure S3. Representative inhibition zone of the non-coated composites against against 5 dif-fererent microorganism.

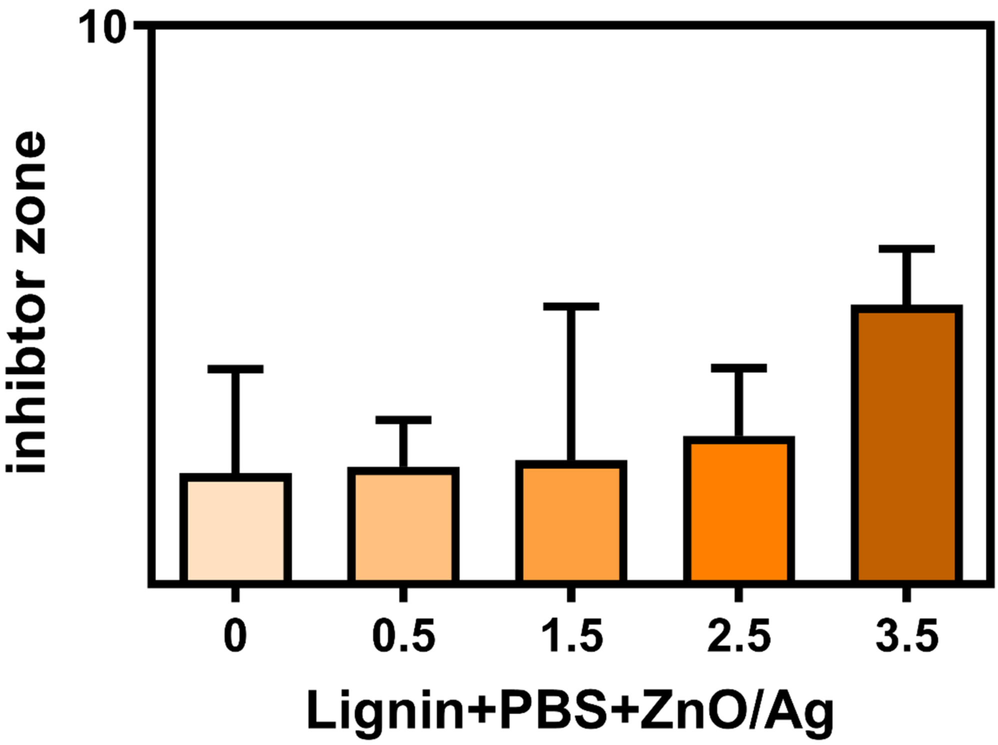

Figure S4. Inhibition zone of the composites with different mass fractions of lignin coated by Ag/ZnO against *Escherichia Coli*. There is no significant difference in the data

© 2022 by the authors. Submitted for possible open access publication under the terms and conditions of the Creative Commons Attribution (CC BY) license (http://creativecommons.org/licenses/by/4.0/).
